# Vasoactive Intestinal Peptide (VIP) in COVID-19 Therapy—Shedding of ACE2 and TMPRSS2 via ADAM10

**DOI:** 10.3390/ijms26062666

**Published:** 2025-03-16

**Authors:** Charlotte Gutzler, Kerstin Höhne, Daniele Bani, Gian Kayser, Sebastian Fähndrich, Michael Ambros, Martin J. Hug, Siegbert Rieg, Valeria Falcone, Joachim Müller-Quernheim, Gernot Zissel, Björn C. Frye

**Affiliations:** 1 Department for Pneumology, University Medical Center, Faculty of Medicine–University of Freiburg, 79106 Freiburg, Germany; 2 Department of Internal Medicine IV, University Hospital Heidelberg, 69120 Heidelberg, Germany; 3 Department of Experimental and Clinical Medicine, Section of Anatomy and Histology, Imaging Platform, University of Florence, 50134 Florence, Italy; 4 Institute of Pathology Naehrig Mattern Kayser, Boetzinger Strasse 60, 79111 Freiburg, Germany; 5 Pharmacy, Medical Center, Faculty of Medicine–University of Freiburg, 79106 Freiburg, Germany; 6 Department of Internal Medicine II, University Medical Center, Faculty of Medicine–University of Freiburg, 79106 Freiburg, Germany; 7 Institute of Virology, University Medical Center, Faculty of Medicine–University of Freiburg, 79106 Freiburg, Germany

**Keywords:** ACE2, vasoactive intestinal peptide, SARS-CoV-2, shedding by ADAM10, TMPRSS2

## Abstract

Patients infected with SARS-CoV-2 may develop mild respiratory symptoms but also Acute Respiratory Distress Syndrome (ARDS). Additionally, severe systemic inflammation contributes to morbidity and mortality. The SARS-CoV-2 virus enters the cell by binding to the angiotensin-converting enzyme 2 (ACE2) receptor, followed by cleavage by transmembrane serine protease 2 (TMPRSS2). Vasoactive intestinal peptide (VIP) is known for its immune-modulating effects by suppressing the release of pro-inflammatory cytokines and enhancing regulatory T-cells. Furthermore, it has been tested in SARS-CoV-2-related clinical trials. We set out to investigate its role in the setting of SARS-CoV-2 infection in vitro. Epithelial cells (CaCo-2) were stimulated with SARS-CoV-2 spike protein, treated with native VIP and analyzed to investigate the mRNA and surface expression of ACE2 and TMPRSS2, the enzyme activity of TMPRSS2 and the infection rate by a SARS-CoV-2 pseudovirus. VIP downregulated ACE2 and TMPRSS2 mRNA and surface expression. Beyond these direct effects, VIP mediates the shedding of surface-expressed ACE2 and TMPRSS2 via upregulation of a sheddase protease (ADAM10). Functionally, these dual mechanisms of VIP-mediated downregulation of proteins involved in SARS-CoV-2 cell entry resulted in a reduced infection rate by the SARS-CoV-2 pseudovirus. These data imply that VIP hampers viral entry mechanisms based on SARS-CoV-2 and the linkage to ADAM10 may stimulate research in other indications beyond SARS-CoV-2.

## 1. Introduction

SARS-CoV-2 causing the COVID-19 pandemic first appeared in 2019 in Wuhan, China [1]. The clinical presentation ranges from mild respiratory complaints to severe Acute Respiratory Distress Syndrome (ARDS) [2,3], leading to more than 6 million deaths [4].

In the initial phase of the pandemic, several therapeutic options have been assessed either to inhibit viral replication or to overcome hyper-inflammation [5,6]. Several drugs have been investigated with initially promising results; however, only a few are currently recommended in the guidelines [7,8].

The SARS-CoV-2 virus enters the cell by binding with its spike protein to a host receptor, namely the ACE2 (angiotensin-converting enzyme 2) receptor [9]. Upon its binding, the virus enters the cell either by clathrin-mediated endocytosis in case of insufficient surface expression of transmembrane serine protease 2 (TMPRSS2) or by membrane fusion. For the latter process, TMPRSS2 is required to allow proteolytic cleavage.

To inhibit SARS-CoV-2 cell entry, several drugs have been assessed, such as antibodies directed against the receptor-binding domain of the SARS-CoV-2 spike protein or ACE2 mimetics, camostat and hydroxychloroquine, which interfere with receptor binding, causing proteolytical inhibition and endosomal activation, respectively.

Membrane proteins such as ACE and TMPRSS2 can undergo the process of shedding, where the ectodomain can be cleaved by proteases called sheddases, e.g., ADAM10 [10,11]. The regulation of ADAM10 is complex and is influenced by miRNAs [12] and various tetraspanins [13,14]. Soluble ACE2 (sACE2), resulting from shedding, changes the susceptibility of cells to SARS-CoV-2 by partially inhibiting virus entry into the cells [15].

Vasoactive intestinal peptide (VIP) was originally described as affecting intestinal fluid and electrolyte transfer [16,17]. Beyond its effects on gastrointestinal functions, VIP influences the vascular tone and the immune response [18,19,20]. It has also been shown that VIP downregulates increased TNF release and increases the induction of regulatory T-cells [21]. Because of its immunomodulatory properties, VIP was investigated in different clinical trials in severe SARS-CoV-2 infection. In 2022 Temerozo et al. indicated a positive correlation between elevated VIP plasma and survival rate in patients with critical COVID-19 [22]. In an initial clinical trial using the intravenous application of VIP, Youssef et al. found a greater survival likelihood from respiratory failure at day 60 in critically ill patients with COVID-19 [23]. Furthermore VIP has been used in different experimental viral infection models, e.g., Vesicular Stomatitis Virus (VSV) [24] and Zika virus [25], showing antiviral potential.

In this study initiated in the beginning phase of the pandemic without established therapies, we set out to understand the effects of VIP on the viral entry mechanisms of SARS-CoV-2 because of its known antiviral and immunomodulatory properties. During the investigation, we found that VIP downregulates the surface expression of SARS-CoV-2 entry machinery-related proteins by reducing mRNA expression and stimulating the ectodomain shedding of utilized receptors via ADAM10.

## 2. Results

### 2.1. Effect of VIP on Epithelial Cells

To unravel the effect of VIP in the setting of SARS-CoV-2, we hypothesized that VIP might influence the inflammatory response of different epithelial cells (e.g., CaCo-2, A549 and BEAS-2B) upon viral infection, which was in vitro assessed by stimulating cells with either a TLR3 (Toll-like receptor 3) agonist (Poly I:C), SARS-CoV-2 spike protein or a combination of both. However, we did not observe significant effects on the mRNA content or the release of pro-inflammatory cytokines or chemokines (e.g., interleukin-6, interferon-γ, CCL2 (CC-chemokine ligand 2) in different epithelial cell lines.

**Figure 1:**
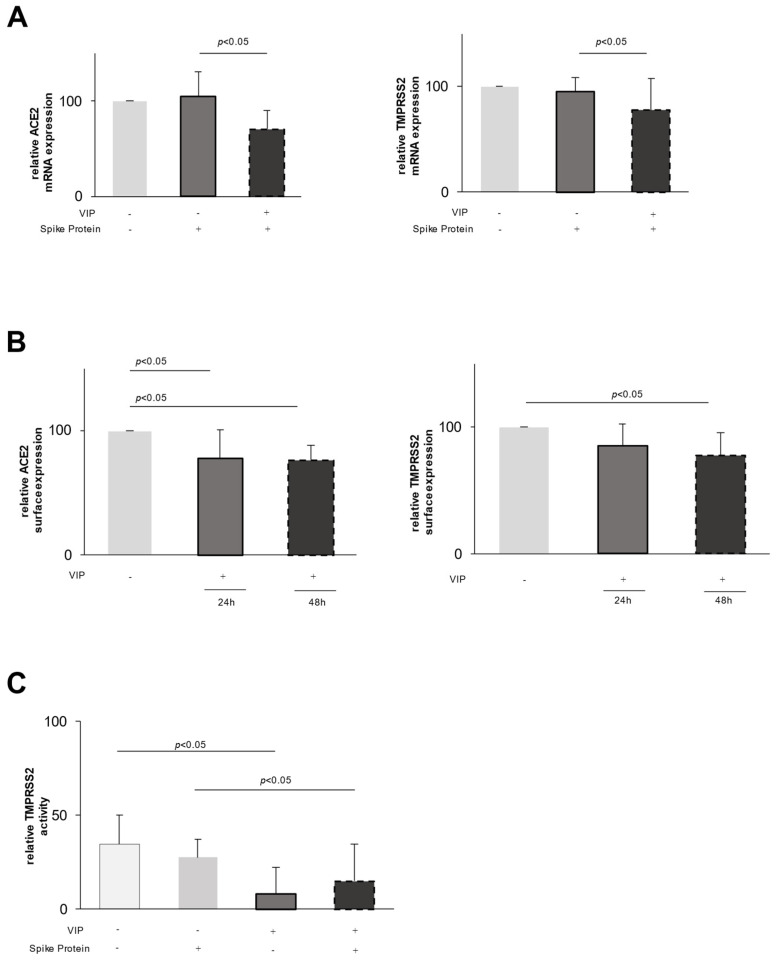
VIP decreases the expression of ACE2 and TMPRSS2 in epithelial cells. (**A**) The mRNA expression of ACE2 and TMPRSS2, measured with qPCR, is significantly reduced in CaCo-2 epithelial cells after stimulation with SARS-CoV-2 spike protein and 24 h incubation with VIP (100 ng/mL) (*n* = 10). Light grey stands for no stimulation, darker grey for stimulation with spike protein and black for stimulation with spike protein and VIP. (**B**) The surface expression of ACE2 and TMPRSS2 was measured in epithelial cells using flow cytometry. VIP (100 ng/mL) significantly reduces ACE2 and TMPRSS2 surface expression at 24 h (*n* = 5) and at 48 h (*n* = 5). Light grey stands for no stimulation. Darker grey for stimulation with VIP for 24 h and black for stimulation with VIP for 48 h. (**C**) VIP (1 µg/mL) significantly reduces the enzymatic activity of TMPRSS2 expressed on the surface (*n* = 5) of both non-stimulated and spike protein-stimulated cells (*n* = 5). The activity change of TMPRSS2 on the surface of the cells was measured with a fluorescence-based assay on vital cells. Lightest grey stands for no stimulation, light grey for stimulation with spike protein, darker grey for stimulation with VIP and black for stimulation with spike protein and VIP.

Interestingly, when assessing the mRNA expression of ACE2 and TMPRSS2, we found a substantial decrease in their respective expression in VIP-exposed epithelial cells. Whereas the stimulation of CaCo-2 cells with the spike protein did not alter the mRNA expression of ACE2 or TMPRSS2 (Figure 1A), the addition of VIP leads to a downregulation of the expression of both ACE2 and TMPRSS2 mRNA (Figure 1A). To extend these findings to the protein level, we used flow cytometry to assess the surface expression of ACE2 and TMPRSS2. In accordance with the mRNA data, VIP decreases the surface expression of both proteins after 48 h (Figure 1B) with a more pronounced effect on ACE2.

To assess the functional relevance, we investigated the protease surface activity of TMPRSS2 using a protease-activated fluorescent peptide. As shown in Figure 1C, upon stimulation of epithelial cells with VIP, there is a significant decrease in the enzymatic activity of TMPRSS2 by 76% (*p* < 0.05, Figure 1C). Similar effects were observed in spike protein-pretreated cells, i.e., a decrease in the enzymatic activity by 37% (*p* < 0.05, Figure 1C).

To evaluate the specificity of the VIP effect, we added two specific VIP antagonists (VIP receptor antagonist and VIP receptor 1 antagonist) to VIP stimulation assays.

**Figure 2:**
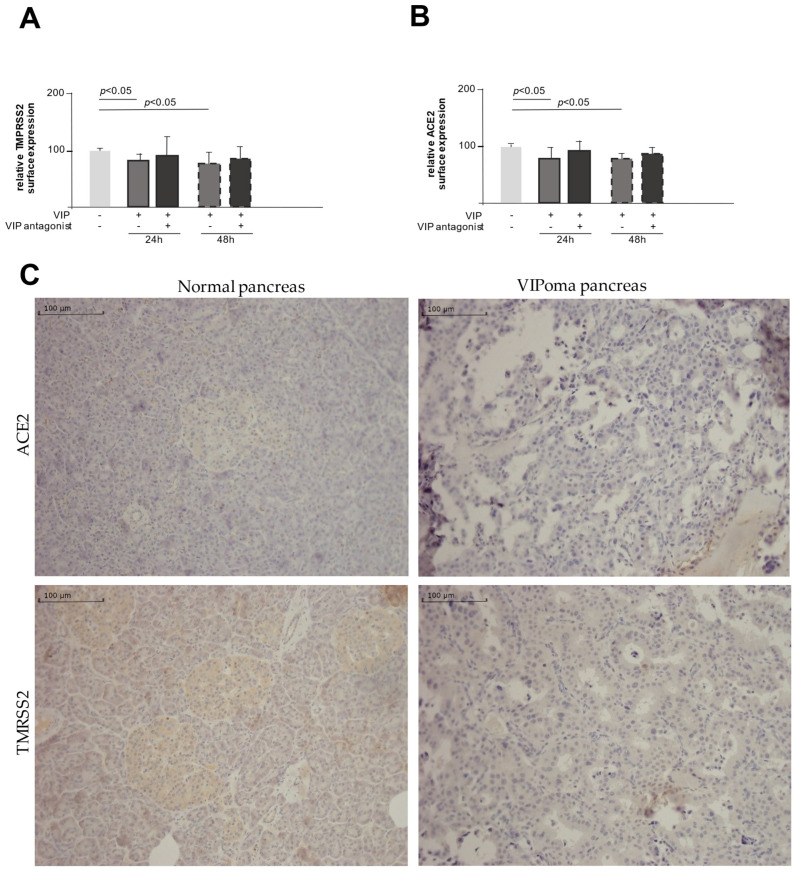
Downregulation of TMPRSS2 and ACE2 expression is blocked by the VIP receptor antagonist. VIP receptor antagonist added together with VIP (100 ng/mL) into the cell culture blocks the VIP-induced downregulation of TMPRSS2 (*n* = 4) (**A**) and ACE2 (*n* = 4) (**B**) expression demonstrating VIP specificity. Lightest grey stands for no stimulation, darker grey for stimulation with VIP and black for stimulation with VIP antagonist. Solid outline stands for stimulation for 24 h and dashed outline for stimulation for 48 h. Tissue from patients with VIP-producing tumors (VIPoma) disclose no expression of ACE2 or TMPRSS2 in the tissue (**C**).

Figure 2A,B clearly demonstrate that the addition of the VIP antagonist abrogates the VIP-induced downregulation of ACE and TMPRSS2 mRNA, emphasizing a specific VIP effect. To further link these in vitro findings to the in vivo situation, we studied qualitatively the expression of TMPRSS2 and ACE2 in the histological samples from two patients with diagnosed VIP-producing tumors and two control samples of pancreatic tissue (Bani Sacchi et al., 1992 [26]). Figure 2C demonstrates on the left the expression of ACE2 and TMPRSS2 in the normal pancreatic tissue, with moderate expression of both proteins. In contrast, the histological expression of ACE2 and TMPRSS2 is markedly reduced in the pancreatic tissue of patients with diagnosed VIPoma.

### 2.2. VIP Mediates TMPRSS2 and ACE2 Shedding via ADAM10

Given the moderate downregulatory effect of VIP on TMPRSS2 and ACE2 mRNA expression compared to the more pronounced effect on the protein surface expression, we speculated that the proteolytic cleavage of expressed proteins may play an additional regulatory role.

**Figure 3:**
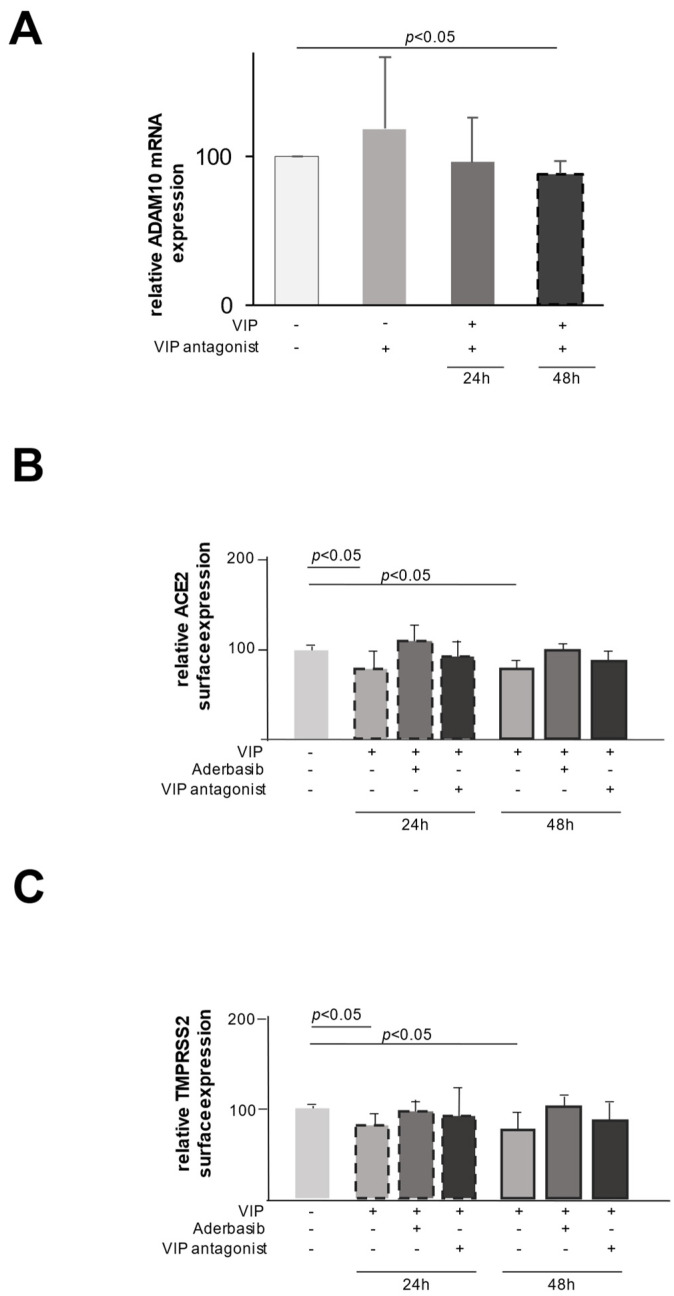
VIP-induced ADAM10 cleaves TMPRSS2 and ACE2, resulting in downregulated surface expression. (**A**) The mRNA expression of ADAM10 was upregulated by VIP stimulation (100 ng/mL) (*n* = 5). This induction was mostly inhibited by the VIP antagonist. At 24 h (*n* = 4) and 48 h (*n* = 4), there is only a slight increase left. Lightest grey stands for no stimulation, light grey for stimulation with VIP antagonist, darker grey for stimulation with VIP and VIP antagonist for 24 h and black for stimulation with VIP and VIP antagonist for 48 h. (**B**) VIP-induced reduction of the surface expression of ACE2 was abrogated by inhibition of ADAM10 by Aderbasib after 24 (*n* = 5) and 48 h (*n* = 5). Lightest grey stands for no stimulation, light grey for stimulation with Aderbasib, darker grey for stimulation with VIP and Aderbasib and black for stimulation with VIP and VIP antagonist. Dashed outline stand for stimulation for 24 h and solid outline for stimulation for 48 h. (**C**) VIP-induced reduction of the surface expression of TMPRSS2 was abrogated by inhibition of ADAM10 by Aderbasib when stimulated for 24 (*n* = 5) and 48 h (*n* = 5). Lightest grey stands for no stimulation, light grey for stimulation with Aderbasib, darker grey for stimulation with VIP and Aderbasib and black for stimulation with VIP and VIP antagonist. Dashed outline stand for stimulation for 24 h and solid outline for stimulation for 48 h.

We, therefore, set out to test the effect of VIP on the expression of different proteases in epithelial cells. As shown in Figure 3A, the stimulation with VIP resulted in an increase in ADAM10 mRNA expression, which was completely abrogated by the addition of a specific VIP antagonist, emphasizing the specific enrollment of VIP. In line with these findings, the addition of a specific ADAM10 inhibitor (Aderbasib) to VIP-stimulated cells counteracts the VIP-mediated shedding of ACE2 and TMPRSS2 and, therefore, the downregulation of the respective surface expression (Figure 3B,C). These findings link the observed VIP-induced upregulation of ADAM10 to its proteolytic role in the process of the shedding of ACE2 and TMPRSS2.

### 2.3. VIP Treatment Reduces the Number of Infected CaCo-2 Cells

**Figure 4:**
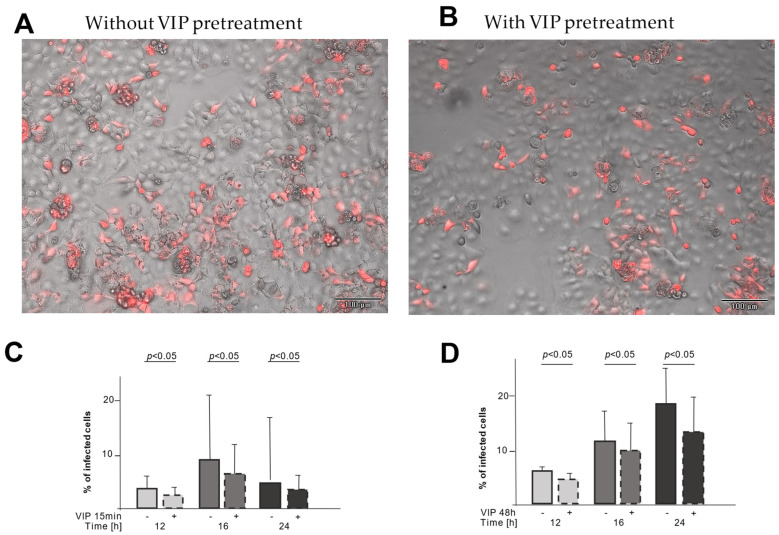
Pretreatment with VIP reduces the infection rate of epithelial cells with a non-pathogenic SARS-CoV-2 pseudovirus. (**A**) An example of a microscopic picture of epithelial cells infected with a SARS-CoV-2 pseudovirus without pretreatment with VIP (10 µg/mL). Only infected cells show a red fluorescence signal. (**B**) Pretreatment with VIP (10 µg/mL) leads to a reduction in the number of infected epithelial cells. The pictures are an overlay of phase contrast and mCherry fluorescent photomicrographs (100× magnification). (**C**) Pretreatment with VIP (10 µg/mL) for 15 min before infection with a SARS-CoV-2 pseudovirus significantly reduces the percentage of infected epithelial cells (12 h *n* = 7, 16 h *n* = 5, 24 h *n* = 9). Light grey stands for stimulation with pseudovirus for 12 h, darker grey for stimulation for 16 h and black for stimulation for 24 h. Solid outline stands for no VIP pretreatment and dashed outline stands for VIP pretreatment (**D**) Pretreatment with VIP (10 µg/mL) for 48 h before infection with a SARS-CoV-2 pseudovirus significantly reduces the percentage of infected epithelial cells (12 h *n* = 6, 16 h *n* = 6, 24 h *n* = 6). Light grey stands for stimulation with pseudovirus for 12 h, darker grey for stimulation for 16 h and black for stimulation for 24 h. Solid outline stands for no VIP pretreatment and dashed outline stands for VIP pretreatment.

To test the functional relevance of this downregulation, we used a non-pathogenic SARS-CoV-2 pseudovirus that uses the same entry mechanism and expresses the red fluorescent mCherry upon cell infection. CaCo-2 cells were infected either directly with the pseudovirus or after preincubation with VIP for either 15 min or 48 h to study the functional relevance of shedding (15 min) and downregulation (48 h) on the viral entry. Exemplary microscopic pictures are shown in Figure 4A (without VIP pretreatment) and B (with VIP pretreatment), demonstrating fewer infected (red fluorescent) cells with VIP pretreatment. For both pretreatment periods, the infection rate was reduced at different time points (Figure 4C,D), enforcing the functional relevance of the ACE2 and TMPRSS2 downregulation.

## 3. Discussion

VIP has been known for its immunomodulatory effects by suppressing the release of pro-inflammatory cytokines and enhancing regulatory T-cells [16,18,19,21], but with respect to SARS-CoV-2 infection, the effect of VIP in vitro and vivo has been unresolved until now.

Therefore, we set out to unravel the potential in vitro mechanism of VIP on epithelial cells in the setting of SARS-CoV-2 and observed an unexpected effect of VIP on the expression of SARS-CoV-2 entry machinery-related proteins.

Our data suggest that VIP may hamper SARS-CoV-2 entry by reducing the surface expression of its cognate entry receptors.

First, VIP downregulates TMPRSS2 and ACE2 expression at a transcriptional level, leading to the reduced surface expression of these proteins (Figure 1). Second, VIP induces the shedding of expressed proteins on the cell surface, potentially related to the increased expression of ADAM10, a metalloprotease involved in TNF (tumor necrosis factor) processing and lung inflammation [27].

Several lines of evidence support these observations. VIP was comparably effective in the downregulation of mRNA and expressed protein levels of ACE2 and TMPRSS2 that could be specifically abrogated by adding a VIP antagonist. In line with these expression data, TMPRSS2 proteolytic function was reduced by VIP linking expression data to functional effects. Furthermore, data obtained from human samples indirectly suggests the in vivo relevance of these in vitro findings. The reduced expression of TMPRSS2 and ACE2 may weaken the binding and reduce the viral entry. Addressing these proteins has been considered an important treatable trait [28,29].

In addition to the expression data, in vitro data suggest that VIP induces protein shedding of TMPRSS2 and ACE2 via the expression of ADAM10 as the shedding of TMPRSS2 and ACE2, and the changes in expression could be blocked by Aderbasib, a known inhibitor of ADAM10.

These data suggest that VIP may hamper SARS-CoV-2 cell entry. This was also demonstrated using a SARS-CoV-2 pseudovirus mimicking SARS-CoV-2 infection. Pre-treatment of cells with VIP reduces the infection rate with the pseudovirus. Whether the treatment with VIP also dampens inflammation, e.g., by the upregulation of ADAM10 and subsequent proteolytic cleavage of proinflammatory cytokines, is speculative and may be addressed in further studies. At least one study suggests that VIP may reduce pro-inflammatory events in monocytes in the setting of SARS-CoV-2 [22]. Of note, the study by Temerozo found a positive association between VIP serum levels and the clinical outcome of SARS-CoV-2 infection.

In addition to its known anti-inflammatory effects, VIP suppresses the surface expression of ACE2 and TMPRSS2, both acting at a transcriptional level and promoting their shedding.

ACE2 and TMPRSS2 are used by the virus to infect the cells. By reducing the mRNA expression of ACE2 and TMPRSS2, VIP weakens the binding of SARS-CoV-2 to the cell. Especially in combination with reducing the surface expression of ACE2 and TMPRSS2 and the reduction of the activity of TMPRSS2, the infection of the cell is impaired by VIP. Corresponding to Zipeto et al., Trougakos et al. and Sharif-Askari et al. described that the severity of COVID-19 correlates with the surface expression of ACE2 and TMPRSS2 [30,31,32].

Up to now, only methods blocking the binding between ACE2/TMPRSS2 and spike proteins and inhibitors of TMPRSS2 and ACE2 have been described. The blocking of TMPRSS2 is an effective method to reduce COVID-19 infection, as shown by Hoffmann et al. [33], by using a clinically proven TMPRSS2 inhibitor (camostat mesylate). Another starting point was the use of an inhibitor that prevents the binding of ACE2 to the spike protein and reduces the infectiousness of SARS-CoV-2 [34].

With our experiments, we were able to show that there are multiple ways in which VIP hampers viral entry mechanisms, including decreasing the gene expression, the surface expression and the activity of the needed receptors.

In addition, we were able to display that VIP induces the shedding of ACE2 and TMPRSS2 from the surface, which contributes to the reduction of the surface expression of these receptors. The emerging soluble ACE2 (sACE2) competes with membrane-bound ACE2 for spike proteins [15] and can potentially be used as a form of trap for the virus. Yeung et al. (2021) showed that lower concentrations of sACE2 are not as effective as high concentrations in reducing cell infection and can even increase the susceptibility of the cells to SARS-CoV-2 [35]. This indicates that the strong stimulation of shedding of ACE2 is needed to reduce the infection of the target cells.

For TMPRSS2, no process of shedding has been described until now.

By measuring the mRNA expression of ADAM10 and using Aderbasib as an ADAM10 inhibitor, we identified ADAM10 as one of the relevant enzymes for the shedding of ACE2 and TMPRSS2. It is important to consider that the regulation of ADAM10 is complex and not only influenced by VIP but also by different miRNAs and the family of tetraspanin [12,14], which may alter the effect of VIP on ADAM10 in different patients and different diseases.

Shedding stimulated by VIP via ADAM10 introduces VIP as a potential therapeutic option for other diseases, e.g., COPD. Leung et al. found that patients with COPD had an upregulation of ACE2, making them more susceptible to COVID-19 and possibly other viral infections [36,37]. Therefore, the downregulation of ACE2 by VIP-induced shedding potentially reduces the risk of viral-induced exacerbations in patients with COPD.

Correlating with our results showing the reduction of viral infection via a pseudovirus, Temerozo et al. [22] showed how VIP decreases SARS-CoV-2 genome replication in monocytes. This indicates that the effects of reducing the mRNA and surface expression of ACE2 and TMPRSS2 are not limited to epithelial cells but can be extended to immune cells as well. The same authors also described how VIP reduces viral replication by inhibiting transcription factors SREBPs and NF-kB, which may be another way viral infection can be downscaled. This indicates that VIP may also have a therapeutic effect on other viral infections by not only changing the receptors used by SARS-CoV-2 but also the replication pathway.

In contrast to Temerezo et al., here, we show a reduction of in vitro cell infection by a SARS-CoV-2 pseudovirus upon VIP pretreatment. It is important to consider that the pseudovirus only exerts one replication cycle, thus only allowing for investigation of the effects of VIP on virus entry by reduced surface receptor expression but not the possible suppression of the spread of infection by inhibiting viral replication. To answer this question, a different cell model with pathogenic SARS-CoV-2 models should be used and may be a matter for future experiments.

Until now, the therapeutic options for inhibiting viral infection and replication and reducing hyperinflammation in COVID-19 and other viral diseases are limited. Our present data show how VIP can hamper viral entry mechanisms in the example of COVID-19. These key findings suggest that VIP may be used in the future for similar purposes as new, promising anti-viral and anti-inflammatory drugs.

## 4. Materials and Methods

### 4.1. Cell Culture and Stimulation Assays

CaCo-2 cells (colorectal adenocarcinoma, 72y, male) were obtained from the cell repository of the Signaling Factory of the University of Freiburg (Freiburg im Breisgau, Germany).

They were cultured in DMEM (Dulbecco’s Modified Eagle Medium) with 10% FCS (fetal calf serum) and 1% Penicillin/Streptomycin (ThermoFisher, Darmstadt, Germany), and 1% of NEA (non-essential aminoacids, Gibco ThermoFisher, Darmstadt, Germany) was added. All cells were grown at 37 °C, in 100% humidity and 5% CO_2_. CaCo-2 cells were used because they are human cells and carry the TMPRSS2 and the ACE2 receptor in a sufficient amount necessary for the subsequent analyses [38,39]. They are highly permissive for SARS-CoV-2 infections [40] and are broadly used in COVID-19 research [41].

For stimulation experiments, the cells were plated at an 80% confluency in 6 or 96-well plates and stimulated with SARS-CoV-2 spike protein (R&D Systems, Inc., Minneapolis, MN, USA) and/or native VIP (100 ng/mL, Bachem, Bubendorf, Switzerland), as indicated in the description of the experiments. Beforehand, dose-finding experiments and time kinetics were performed. To assess the specificity of the VIP effect, two specific and competitive VIP antagonists were used: (VIP receptor antagonist (His-Ser-Asp-Ala-Val-4-Cl-D-Phe-Thr-Asp-Asn-Tyr-Thr-Arg-Leu-Arg-Lys-Gln-Leu-Ala-Val-Lys-Lys-Tyr-Leu-Asn-Ser-Ile-Leu-Asn-NH2) and VIP receptor 1 antagonist (Ac-His-D-Ph)-Asp-Ala-Val-Phe-Thr-Asn-Ser-Tyr-Arg-Lys-Val-Leu-Lys-Arg-Leu-Ser-Ala-Arg-Lys-Leu-Leu-Gln-Asp-Ile-Leu-NH2) (Phoenix Pharmaceuticals, Inc, CA, USA).

### 4.2. Harvesting Cells with RNA Isolation Kit from ExtractMe^®^

Cells were harvested 24 h or 48 h after stimulation and resuspended in lyse buffer prior to RNA isolation using the RNA Isolation Kit from ExtractMe^®^ (Bioscience GmbH, Neuenburg, Germany) according to the manufacturer’s instructions. The RNA concentration was determined using the NanoDrop 2000c spectrophotometer (ThermoFisher, Darmstadt, Germany) and the associated software (v1.6.198).

### 4.3. Real-Time PCR for mRNA Expression

The mRNA was transcribed into complementary DNA (cDNA) using the iScript^TM^ cDNA Synthesis Kit (BioRad Laboratories GmbH, Hercules, CA, USA) according to the protocol provided. The relative expression of the targets in relation to the expression of GAPDH (Glyceraldehyde-3-phosphate dehydrogenase) was determined with quantitative real-time PCR. After transcription, the DNA was diluted 1:10 and the relative expression of our target genes was estimated using SYBR-Green^®^ (BioRad Laboratories GmbH, Hercules, CA, USA) and a CFX96 PCR machine (BioRad Laboratories GmbH, Hercules, CA, USA). The relative expression (rE) of the target gene was calculated using a modified delta-delta-Ct method (ΔΔCtmethod):

$$rE=\left(2^{\left(cT\left(GAPDH\right)-cT(targetgene\right)}\right)\times 10000$$

The primers that were used for PCR are depicted in Table 1.

### 4.4. Flow Cytometry

After stimulation, the cells were detached with trypsin and then fixed for 5 min with 4% PFA (paraformaldehyde), washed two times with PBS and then incubated for 15 min in 1% FCS and human (h)IgG to prevent unspecific binding. Direct immune staining of targeted antigens was achieved by incubation with anti-TMPRSS2 and anti-ACE2 antibodies for 20 min at room temperature. The respective isotype controls were included. Cells were measured on a Cytoflex (Beckman Coulter, Life Sciences, Brea, CA, USA) and analyzed using CyteExpert (Beckman Coulter Life Sciences, Brea, CA, USA). Antibodies used for flow cytometry are shown in Table 2.

### 4.5. Fluorescence-Based Assay for Measuring TMPRSS2 Activity

A fluorescent peptide (Boc-Gln-Ala-Arg-MCA, PeptaNova, Sandhausen, Germany) was used as a substrate for TMPRSS2. Upon proteolytic cleavage by TMPRSS2, the fluorescence wavelength changes. Cells were incubated in a black 96-well plate and stimulated with 100 ng/mL SARS-CoV-2 spike protein, 1 µg/mL VIP or both for 24 h. Next, cells were washed with PBS. After that, the buffer (Tris 50 mM, NaCl 150 mM, pH = 8) containing 50 nM fluorescent peptide was added. As a blank, only buffer without the fluorescent peptide was used and later subtracted.

Fluorescence was measured with EnVision Multiplate Reader (PerkinElmer, Hopkinton, MA, USA) at 340 nm excitation and 440 nm emission. To compare the values at 0 min (T0) and 30 min (T30), the following formula was used:

$$increase\;in\;\%=\frac{100}{T0\times T30}-100$$

### 4.6. Immunohistochemistry

All tissue specimens were formalin-fixed and paraffin-embedded via routine procedures. Five µm-thick tissue sections of the normal pancreas as well as pancreatic VIPoma were subjected to immunohistochemical stainings with ACE2 (Santa Cruz Biotechnology, Inc., Dallas, TX, USA; dilution 1:100) and TMPRSS2 (Santa Cruz Biotechnology; dilution 1:50). All staining protocols were established on the DAKO link Autostainer Platform (DakoCytomation, Agilents, Glostrup, Denmark) with DAKO EnVision+ Dual Link System HRP (K4065) for antibody detection and visualization. Finally, the slides were counterstained with hematoxilin.

### 4.7. Measuring SARS-CoV-2 Pseudovirus Infection Rate in Cell Cultures

Adherent cells were infected with SARS-CoV-2 pseudovirus (Pseudotyped VSV-SARS-CoV-2 SΔG-mCherry, Creative Diagnostics, Shirley, NY, USA). The virus undergoes only one replication cycle and codes the fluorescent protein “mCherry”. If the cells are infected, mCherry is expressed and can be detected via fluorescence microscopy. Cells were incubated for either 15 min or 48 h with VIP before the infection with the pseudovirus. After 12, 16 and 24 h, the infection rate was measured using automatic fluorescence microscopy (EVOS™ M7000 Imaging System, ThermoFisher, Darmstadt, Germany). For analysis, 65% of the cell culture surface of each well was scanned automatically in 200 pictures using phase contrast microscopy and fluorescence microscopy.

As a positive control, EVUSHELD^®^ (Tixagevimab/Cilgavimab, 10 µg/mL, AstraZeneca, Cambridge, UK) was added to the cell cultures in the same time pattern as VIP.

The pictures were analyzed with the EVOS Analysis application “Celleste” using the “transfection efficiency” tool. The percentage of infected cells (i.e., mCherry-positive cells) were set in relation to the whole cell population in the area analyzed. For each picture, the confluence and infection rate were estimated and the ratio was calculated. For analysis per well, the mean and standard deviation from these results were computed.

### 4.8. Data Presentation and Statistics

Data are shown as the mean value of biological replicates, with error bars depicting the standard deviation. The paired analysis was conducted via a *t*-test with STATVIEW (version 5.01; SAS Institute, Cary, NC, USA). The number of biological replicates is shown in figure legends. In all applied statistical tests, *p*  ≤  0.05 was regarded as significant, and the figures were labeled accordingly.

## Figures and Tables

**Table 1:** Primers used for PCR. The table depicts the primer name, the accession number of the sequences used to develop the primer and the primer sequences, as well as the melting temperature. The primers were developed using AmplifX© and synthesized by Biomers, Ulm, Germany.

Primer	Accession Number	Sequence	Tm (°C)
GAPDH_LISP	NM_002046.7	5′-CAC CAG GGC TGC TTT TAA CT-3′	55
GAPDH_RISP		5′-GAT CTC GCT CCT GGA AGA TG-3′	54
hsACE2	NM_001371415	5′-GCC CTC TGC ACA AAT GTG ACA TCT-3′	59
hsACE2		5′-TTT CCA ATG CTA GGG TCC AGG GTT-3′	61
hsTMPRSS2	NM_001135099.1	5′-CTG CAG GGA CAT GGG CTA TAA GAA-3′	58
hsTMPRSS2		5′-GAT ATC GAC ATT GCC GGC ACT T-3′	58
hsADAM10	NM_001110.4	5′-TGG TGC TCA TGT ACC TCC CAA A-3′	56
hsADAM10		5′-GGT GTG CAC TCT GTT CCA GAA TCA-3′	58

**Table 2:** Antibodies used for flow cytometry. The table depicts the names of the antibodies and the suppliers, as well as the dilution used for staining.

Antibody	Supplier	Dilution
mouse anti-hACE2 AlexaFluor647	R&D Systems, Inc., Minneapolis, MN, USA	1:30
mouse anti-TMPRSS2-FITC	Santa Cruz Biotechnology, Inc., Dallas, TX, USA	1:30
mouse IgG1 FITC	ImmunoTools, Friesoythe, Germany	1:30
mouse IgG2a AlexaFluor647	Santa Cruz Biotechnology, Inc., Dallas, TX, USA	1:30

## Data Availability

This article is a revised and expanded version of an abstract entitled “Vasoactive intestinal peptide (VIP) suppresses ACE2- and TMPRSS2 expression in stimulated epithelial cells”, which was presented at the European Respiratory Society International Congress, Barcelona, Spain, 6 September 2021 [42].
